# Effect of differences in extubation timing on postoperative care following abdominal aortic replacement surgery: a comparison study

**DOI:** 10.1186/s12871-015-0027-7

**Published:** 2015-03-31

**Authors:** Naomi Ono, Junko Nakahira, Toshiyuki Sawai, Yosuke Kuzukawa, Toshiaki Minami

**Affiliations:** Department of Anesthesiology, Osaka Medical College, 2-7 Daigaku-machi, Takatsuki, Osaka 569-8686 Japan

**Keywords:** Abdominal aortic replacement surgery, Timing of extubation, Cost in intensive care unit

## Abstract

**Background:**

Abdominal aortic replacement requires an extensive incision and strict blood pressure control, making rapid extubation of the tracheal tube and pain management difficult. The effects of extubation timing on the postoperative course and medical costs in the intensive care unit (ICU) were analyzed.

**Methods:**

Patients who underwent elective abdominal aortic replacement were evaluated retrospectively. Patients were divided into those extubated on the day of surgery (Group A) and those extubated later (Group B). Group A was subdivided into extubation in the operating room (Group A1) or in the ICU (Group A2). Intubation time in the ICU, postoperative ICU stay, hospital stay, and total ICU expenses were compared among the four groups.

**Results:**

Of the 191 patients, 95 were extubated on the day of surgery (Group A) and 96 later (Group B). The two groups differed in age and percutaneous coronary intervention history. Surgery and anesthesia durations, intraoperative infusion volume, and intraoperative bleeding amounts differed significantly in the two groups. Epidural anesthesia was given more frequently in Group A. Mean intubation time in the ICU (2.6 ± 2.8 vs 17.4 ± 5.1 hours, P < 0.01), the ICU stay (2.1 ± 0.3 vs 2.4 ± 0.8 days, P < 0.01), and the hospital stay (16.4 ± 5.2 vs 20.2 ± 12.5 days, P = 0.02) were significantly shorter, and total ICU expenses were significantly lower (1,036 ± 307 vs 1,565 ± 1,072 dollars, P < 0.01), in Group A than in Group B. Of the 95 patients in Group A, 34 were extubated in the operating room (Group A1) and 61 in the ICU (Group A2). Arrhythmia, epidural anesthesia, and the amount of intraoperative infusion amount were significantly higher, and the percentage of women significantly lower, in Group A1 (vs Group A2). Postoperative ICU and hospital stays and the ICU costs were not significantly different.

**Conclusion:**

Tracheal tube extubation on the day of abdominal aortic replacement surgery resulted in better postoperative course and lower costs than when extubation occurred later. Patients extubated in the operating room or the ICU on the day of surgery had similar postoperative courses and costs.

## Background

Abdominal aortic replacement surgery requires an extensive incision and strict control of blood pressure, making rapid extubation of the tracheal tube and pain management difficult. The use of anticoagulants makes it difficult to obtain patient informed consent of epidural anesthesia [1]. Following surgery, patients are admitted to an intensive care unit (ICU). If their postoperative course in the ICU is uneventful, they are moved to a ward.

Proper timing of extubation of the tracheal tube after abdominal aortic replacement surgery may benefit patients and reduce the need for subsequent emergency surgery. This retrospective observational study was designed to evaluate the effects of the timing of extubation after postoperative ICU admission and later during the hospital stay, as well as the medical costs of the ICU length of stay (LOS).

## Methods

After obtaining approval from the Ethics Committee of Osaka Medical College (approval number: 1234), data on patients who underwent abdominal aortic replacement surgery from January 2007 to December 2012 were reviewed retrospectively. Patients who underwent surgery with an aortic cross clamp under the renal arteries were included, whereas those who underwent emergency surgery or in whom an I-shaped graft was used were excluded.

All patients received general anesthesia and underwent epidural catheterization on the day before surgery. Epidural anesthesia was omitted in patients with a coagulopathy (international normalized ratio of prothrombin > 1.5, partial thromboplastin time > 45 seconds, platelet count < 100,000/mm^3^), those who took thrombolytic or anti-platelet drugs within 1 week before surgery, and those with myocardial ischemia. Epidural anesthesia was also not administered to patients with lumbar spine deformation, a history of lumbar or thoracic spine surgery, or numbness of the lower extremities due to lumbar or thoracic spine deformation. On the day before surgery, using an 18G Tuohy needle (Smith Medical, Tokyo, Japan), a catheter for epidural anesthesia was inserted into the epidural space at a site between Th8/9 and L1/2. Next, 3 ml of 1% mepivacaine was administered through the epidural catheter to confirm the abnormality. The epidural catheter was left in place for a maximum of 7 days.

General anesthesia was induced by inhalation of 3% sevoflurane and intravenous administration of vecuronium 0.1 mg/kg, fentanyl 0.1 mg/kg, and propofol 1.5–2.0 mg/kg. It was maintained by inhalation of sevoflurane and intravenous administration of rocuronium. Analgesia during surgery consisted of continuous intravenous administration of remifentanil 0.25–0.75 μg/kg/min in patients without epidural anesthesia or continuous epidural administration of 0.375% ropivacaine 5 ml/hr in patients with epidural anesthesia. Throughout the operation, we monitored the patient’s electrocardiogram, arterial pressure, central venous pressure (with an internal jugular vein catheter), percutaneous oxygen saturation, urine volume, capnography, and the bispectral index. Systolic blood pressure was adjusted to 75–90 mmHg during surgery using intravenous remifentanil, alprostadil, nicardipine, and/or calcium, and/or by controlling the infusion volume. Before the aortic cross clamp was applied, intravenous heparin was administered to increase the activated clotting time to > 250 seconds. Blood transfusions were administered as necessary to maintain the hemoglobin concentration at ≥ 9.0 g/dl. Postoperative analgesia consisting of a continuous infusion of 0.2% ropivacaine 5 ml/hr in patients with epidural catheters or intravenous fentanyl 0.25–1.0 μg/kg/hr to patients without epidural catheters, were administered until the patients were admitted to the wards. We did not use patient-controlled epidural anesthesia or patient-controlled IV infusions. Nor did we use a VAS (visual analog scale) to evaluate postoperative pain. Doctors working in the ICU manage pain with intravenous pain medicine and epidural anesthesia.

Tracheal extubation criteria included a response to verbal commands, body temperature ≥ 35.5°C, arterial oxygen partial pressure ≥ 70 mmHg, arterial carbon dioxide partial pressure < 50 mmHg, and respiratory rate of 10–20 breaths/min in accordance with airway management guidelines at our institution. We did not monitor neuromuscular parameters. Beginning in January 2011, we began to use sugammadex for extubation in the operating room, although neostigmine was used for most patients. After the procedure, all patients were admitted to the ICU, with or without intubation.

Patients were divided into two groups, those extubated on the day of surgery (Group A) and those extubated on subsequent days (Group B). Patients in Group A were further subdivided into those extubated in the operating room (Group A1) and those extubated in the ICU (on the day of surgery) (Group A2). The postoperative ICU LOS, total medical expenses in the ICU, postoperative hospital LOS, death within 2 years, and postoperative complications were investigated and compared in Groups A and B and in Groups A1 and A2. Total medical expenses in the ICU included per-patient costs for blood tests, imaging, blood transfusion and infusion, and all other consumables (e.g., suction tubes, syringes). Total intubation time was defined as the time (in hours) from admission to the ICU to the time of extubation in the ICU. Patients extubated in the operating room had an intubation time of 0 hours.

Between-group comparisons were evaluated using the Mann–Whitney U-test, Student’s t-test, Fisher’s extact test, and the χ^2^ test, as applicable. A value of P < 0.05 was considered to indicate statistical significance. All analyses were performed using GraphPad Prism version 5.0 for Mac (GraphPad Software, San Diego, CA, USA).

## Results

Of the 191 patients included in this study, one patient who was extubated in the ICU on the day of surgery died in hospital because of postoperative recurrent pneumonia. In addition, eight patients experienced pneumonia or atelectasis, three developed postoperative renal function disorders, one had postoperative ileus, and one had ischemic enteritis. One patient required surgery under general anesthesia to remove an arterial blood clot just after the abdominal aortic replacement surgery.

Of the 191 patients, 95 were extubated on the day of surgery (Group A) and 96 on subsequent days (Group B). Age and a medical history of percutaneous coronary intervention differed significantly in these two groups (Table 1). Analysis of intraoperative parameters showed significant between-group differences regarding the operating time, duration of anesthesia, intraoperative infusion volume, and amount of intraoperative bleeding. Group A patients were given epidural anesthesia more frequently than those in Group B. The mean intubation time in the ICU (2.6 ± 2.8 vs 17.4 ± 5.1 hours, P < 0.01), mean ICU LOS (2.1 ± 0.3 vs 2.4 ± 0.8 days, P < 0.01), and mean postoperative hospital LOS (16.4 ± 5.2 vs 20.2 ± 12.5 days, P = 0.02) were significantly shorter in Group A. Hospital stay exceeded 30 days in 10 patients because of a respiratory disorder, postoperative ileus, or ischemic enteritis. There were no complications associated with the epidural anesthesia and no major cardiovascular events, such as stroke or myocardial infarction. The total per-patient medical expenses in the ICU were significantly lower in Group A than in Group B (1,036 ± 307 vs 1,565 ± 1,072 dollars; P < 0.01) (Table 2). We converted yen into US dollars at a rate of 113.6 yen per dollar.

**Table 1:** **Baseline demographic and clinical characteristics of patients who underwent abdominal aortic replacement surgery and were extubated on the day of surgery (Group A) or on subsequent days (Group B)**

	Group A (n = 95)	Group B (n = 96)	*P*value
Age (years)	69±9	73±7	0.004
Height (cm)	164.4±7.8	164.5±7.7	0.939
Weight (kg)	60.3±8.8	62.9±12.5	0.166
Female	11 (11.6%)	12 (12.5%)	0.845
Medical history			
Hypertension	63 (66.3%)	73 (76.0%)	0.138
Diabetes mellitus	20 (21.1%)	23 (24.0%)	0.631
Hyperlipidemia	21 (22.1%)	26 (27.1%)	0.425
Chronic obstructive pulmonary disease	7 (7.4%)	4 (4.2%)	0.372
Interstitial pneumonia	1 (1.1%)	2 (2.1%)	1.000
Asthma	4 (4.2%)	1 (1.0%)	0.211
Angina pectoris	9 (9.5%)	16 (16.7%)	0.141
Previous cardiac surgery	14 (14.7%)	16 (16.7%)	0.714
Coronary intervention	15 (15.8%)	28 (29.2%)	0.027
Arrhythmia	6 (6.3%)	7 (7.3%)	0.789
Cardiac disease	2 (2.1%)	3 (3.1%)	1.000
Hemodialysis	2 (2.1%)	1 (1.0%)	0.621
Medication			
Calcium channel blocking agent	45 (47.4%)	56 (58.3%)	0.129
β receptor blocker	17 (17.9%)	18 (18.8%)	0.879
ARB/ACEI	43 (45.3%)	48 (50.0%)	0.512
Statin	20 (21.1%)	21 (21.9%)	0.890

Data are expressed as mean ± standard deviation or as number (%).

*Abbreviations:ARB* angiotensin II receptor blocker; *ACEI* angiotensin-converting enzyme inhibitor.

**Table 2:** **Postoperative outcomes in patients who underwent abdominal aortic replacement surgery and were extubated on the day of surgery (Group A) or on subsequent days (Group B)**

	Group A (n = 95)	Group B (n = 96)	*P* value
Operating time (min)	271 ± 60	313 ± 85	0.001
Anesthetic time (min)	378 ± 61	424 ± 85	< 0.001
Intraoperative parameters			
Infusion volume (ml)	4169 ± 1125	4908 ± 1507	< 0.001
Transfusion	38 (40.0%)	48 (50.0%)	0.165
Urinary volume (ml)	729 ± 559	741 ± 551	0.923
Amount of bleeding (ml)	711 ± 459	900 ± 641	0.017
Mesenteric traction syndrome	7 (7.4%)	14 (14.6%)	0.111
Final ACT	147 ± 18	152 ± 24	0.280
Epidural anesthesia	33 (34.7%)	12 (12.5%)	< 0.001
ICU-related parameters			
Albumin volume	177 ± 379	338 ± 427	< 0.001
Transfusion	5 (5.3%)	12 (12.5%)	0.079
Postoperative complications			
Respiratory disorders	2 (2.1%)	8 (8.3%)	0.100
Renal dysfunction	1 (1.1%)	2 (2.1%)	1.000
Cognitive dysfunction	0 (0.0%)	1 (1.0%)	1.000
Ileus	0 (0.0%)	1 (1.0%)	1.000
Ischemic enteritis	0 (0.0%)	1 (1.0%)	1.000
Thrombectomy	1 (1.1%)	0 (0.0%)	0.497
In-hospital death	1 (1.1%)	0 (0.0%)	0.479
Postoperative intubation time (hr)	2.6 ± 2.8	17.4 ± 5.1	< 0.001
ICU stay (days)	2.1 ± 0.3	2.4 ± 0.8	< 0.001
Postoperative hospital stay (days)	16.4 ± 5.2	20.2 ± 12.5	0.017
Total expense in ICU (dollars)	1,036 ± 307	1,565 ± 1,072	<0.001
First day expense in ICU (dollars)	637 ± 37	641 ± 44	0.012
Last day expanse in ICU (dollars)	352 ± 183	422 ± 213	0.002

Data were expressed as mean ± standard deviation or as number (%). *Abbreviations:ACT* activated clotting time; *ICU* intensive care unit.

Of the 95 patients extubated on the day of surgery, 34 were extubated in the operating room (Group A1) and 61 in the ICU (Group A2). The percentages of women and patients with arrhythmia were significantly higher in Group A2. Epidural anesthesia was performed more frequently in Group A1 than in Group A2. The intraoperative infusion volume was greater in Group A1 than in Group A2 (Table 3). There were no significant differences in postoperative care, including postoperative ICU LOS, hospital LOS, or ICU costs (Table 4).

**Table 3:** **Baseline demographic and clinical characteristics of patients who underwent abdominal aortic replacement surgery and were extubated on the day of surgery in the operating room (Group A1) or in the ICU (Group A2)**

	Group A1 (n = 34)	Group A2 (n = 61)	*P*value
Age (years)	69 ± 8	69 ± 10	0.686
Height (cm)	164.4 ± 6.1	164.4 ± 8.6	0.993
Weight (kg)	61.1 ± 8.6	59.8 ± 8.9	0.510
Female	0 (0.0%)	11 (18.0%)	0.007
Medical history			
Hypertension	21 (61.8%)	42 (68.9%)	0.484
Diabetes mellitus	5 (14.7%)	15 (24.6%)	0.257
Hyperlipidemia	7 (20.6%)	14 (23.0%)	0.790
Chronic obstructive pulmonary disease	4 (11.8%)	3 (4.9%)	0.245
Interstitial pneumonia	0 (0.0%)	1 (1.6%)	1.000
Asthma	1 (2.9%)	3 (4.9%)	1.000
Angina pectoris	5 (14.7%)	4 (6.6%)	0.274
Previous cardiac surgery	3 (8.8%)	11(18.0%)	0.366
Coronary intervention	8 (23.5%)	7 (11.5%)	0.123
Smoking	15 (44.1%)	26 (42.6%)	0.888
Arrhythmia	5 (14.7%)	1 (1.6%)	0.021
Cardiac disease	1 (2.9%)	1 (1.6%)	1.000
Hemodialysis	0 (0.0%)	2 (3.3%)	0.536
Medication			
Calcium channel blocking agent	18 (52.9%)	27 (44.3%)	0.417
β receptor blocker	10 (29.4%)	7(11.5%)	0.029
ARB/ACEI	13 (38.2%)	30 (49.2%)	0.304
Statin	7 (20.6%)	13 (21.3%)	0.934

Data were expressed as mean ± standard deviation or as number (%).

*Abbreviations:ARB* angiotensin II receptor blocker; *ACEI* angiotensin-converting enzyme inhibitor; *ICU* intensive care unit.

**Table 4:** **Postoperative outcomes in patients who underwent abdominal aortic replacement surgery and were extubated on the day of surgery in the operating room (Group A1) or in the ICU (Group A2)**

	Group A1 (n = 34)	Group A2 (n = 61)	*P*value
Operating time (min)	267 ± 56	274 ± 63	0.592
Anesthetic time (min)	374 ± 53	380 ± 66	0.696
Intraoperative parameters			
Infusion volume (ml)	4512 ± 1229	3978 ± 1023	0.041
Transfusion	16 (47.1%)	22 (36.1%)	0.294
Urinary volume (ml)	839 ± 642	668 ± 502	0.319
Amount of bleeding (ml)	699 ± 481	718 ± 450	0.698
Mesenteric traction syndrome	2 (5.9%)	5 (8.2%)	1.000
Final ACT	147 ± 18	148 ± 18	0.908
Epidural anesthesia	17 (50.0%)	16 (26.2%)	0.020
ICU-related parameters			
Albumin volume (ml)	129 ± 380	203 ± 379	0.147
Transfusion	1 (2.9%)	4 (6.6%)	0.652
Postoperative complications			
Respiratory disorders	0 (0.0%)	2 (3.3%)	0.536
Renal dysfunction	1 (2.9%)	0 (0.0%)	0.358
Thrombectomy	1 (2.9%)	0 (0.0%)	0.358
In-hospital death	0 (0.0%)	1 (1.6%)	1.000
Postoperative intubation time (hr)	0	4.0 ± 2.6	NA
ICU stay (days)	2.0 ± 0.2	2.1 ± 0.3	0.647
Postoperative hospital stay (days)	15.8 ± 5.1	16.7 ± 5.3	0.496
Total expense in ICU (dollars)	1,003 ± 272	1,054 ± 326	0.813
First day expense in ICU (dollars)	645 ± 52	633 ± 23	0.700
Last day expense in ICU (dollars)	337 ± 166	361 ± 193	0.563

Data were expressed as mean ± standard deviation or as number (%).

*Abbreviations:ACT* activated clotting time; *ICU* intensive care unit.

## Discussion

This retrospective study was designed to investigate the optimal timing of postoperative extubation in patients who underwent abdominal aortic replacement surgery, as shown by the postoperative ICU LOS, hospital LOS, and ICU costs. We found that the postoperative ICU LOS and hospital LOS were significantly shorter, and medical costs significantly lower, in patients extubated on the day of surgery than in those extubated later. There were no significant differences in the ICU expenses, postoperative ICU LOS, or hospital LOS, however, between patients extubated in the operating room and those extubated in the ICU on the day of surgery. These findings indicate that extubation on the day of surgery may benefit patients undergoing abdominal aortic replacement surgery by reducing medical expenses and the overall hospital LOS. Fewer female patients were extubated on the day of surgery because of differences between the sexes regarding the severity of the abdominal aortic aneurysm [2,3]. As has been reported elsewhere, female patients in this study who underwent this operation had more severe postoperative conditions than the male patients.

Epidural anesthesia was found to facilitate early postoperative extubation. In the present study, younger age and fewer interventions enabled the performance of epidural anesthesia, which in turn caused the anesthesiologists to extubate these patients earlier. A study of patients undergoing general surgery was unable to determine whether epidural anesthesia affected postoperative outcomes (e.g., the postoperative hospital LOS) [4,5], although epidural anesthesia has been reported to have an analgesic effect [6,7]. In addition, patient satisfaction was higher when epidural anesthesia was combined with general anesthesia [8]. Although the addition of epidural anesthesia has been found to provide a postoperative analgesic effect and to maintain postoperative respiratory function in patients undergoing abdominal aortic replacement surgery, it had no effect on patient outcomes [9,10].

We found that the mean duration of maintaining epidural anesthesia was about 3 days, including the day of surgery. The effects of epidural analgesia in patients undergoing abdominal aortic replacement surgery have been reported to be significant only on the first day after surgery, with almost no effect on days 3–7 [11]. We therefore regarded removing the epidural catheter 3 days after surgery as appropriate.

Complications associated with epidural anesthesia are hematoma and infection. Because perioperative anticoagulation therapy is becoming more complicated, few patients who underwent abdominal aortic replacement surgery in our hospital in 2014 were given epidural anesthesia. Our postoperative goal for patients undergoing abdominal aortic replacement surgery is rapid extubation in the ICU, followed by an optimal analgesic method consisting of systemic analgesia and a peripheral nerve blocker.

We found that the amount of infusion was significantly greater in patients extubated later than on the day of surgery because they had had significantly longer anesthesia and operating times. The amount of the infusion, however, was greater in patients extubated in the operating room than in those extubated in the ICU on the day of surgery—despite there being no significant differences in their anesthesia or operative durations. However, a significantly higher percentage of patients extubated in the operating room had been given epidural anesthesia. The amount of the infusion was greater in these patients to prevent hypotension due to the blocked sympathetic system.

This study had several limitations. First, analysis on a case-by-case basis may have influenced our results, especially in regard to postoperative analgesia, because additional postoperative analgesia depended on evaluations by intensivists. Second, although this study included patients older than 70 years, we did not evaluate postoperative cognitive dysfunction because none of these patients required prolonged ICU stay due to postoperative cognitive dysfunction. Third, because the most important goal was to avoid fatal postoperative complications, the timing of the extubation tended to be longer. Fourth, we did not consider the effects of sugammadex, which was used in some cases for extubation in the operating room.

## Conclusions

Extubation on the day of surgery was advantageous for patients as it reduced medical expenses and shortened the postoperative hospital LOS. Extubation in the operating room did not prove advantageous. Because epidural anesthesia could shorten the extubation time, alternative analgesic methods are required. Our study results recommend extubation in the ICU, especially on the day of surgery.
